# Patterns and Associations of Physical Activity, Screen Time, Sleep, and Dietary Habits among Saudi Females Participating in Fitness Centers

**DOI:** 10.3390/healthcare10060958

**Published:** 2022-05-24

**Authors:** Mezna A. AlMarzooqi, Nada M. Albawardi, Abeer A. Altamimi, Arwa S. Altalhi, Hazzaa M. Al-Hazzaa

**Affiliations:** 1 Department of Community Health Sciences, College of Applied Medical Sciences, King Saud University, Riyadh 11451, Saudi Arabia; 2 Lifestyle and Health Research Center, Health Sciences Research Center, Princess Nourah Bint Abdulrahman University, Riyadh 11451, Saudi Arabia; nbwardi@yahoo.com (N.M.A.); arsaltalhi@pnu.edu.sa (A.S.A.); halhazzaa@hotmail.com (H.M.A.-H.); 3 Health Sciences Research Center, Princess Nourah Bint Abdulrahman University, Riyadh 84428, Saudi Arabia; aaaltamimi@pnu.edu.sa

**Keywords:** dietary habits, women, fitness center, physical activity, Saudi Arabia, sedentary behaviors, sleep duration

## Abstract

This study was designed to investigate the level and associations of physical activity, sedentary behavior, sleep, and dietary habits among Saudi women attending fitness centers in Riyadh. A descriptive cross-sectional study was carried out from 12 fitness centers in Riyadh, Saudi Arabia. A total of 460 participants answered a two-part survey self-administered questionnaire, which included information about the demographic characteristics and lifestyle. The analysis found significant differences between the two groups in terms of age, weight, BMI, and combined prevalence of being overweight and obese. Compared with less active females, high active females showed significantly more favorable dietary habits (*p* < 0.05). Significantly higher values were observed for the total physical activity energy expenditure in METs-min/week (*p* = 0.028). Moreover, females with high screen time (>3 h per day) were younger, less active, slept longer, and had higher intakes of fast foods and chocolates/candy intake (*p* = 0.001). Overall, the participants were highly active, exceeding the recommended physical activity needed to enhance health. The analysis also showed sufficient sleep duration (>7 h/night) was significantly associated with the diet and nutrition of the participants. Health education programs are needed to reduce the risks of sedentary behavior, sleep problems, and dietary habits.

## 1. Introduction

Saudi Arabia has undergone remarkable growth in the last few decades at an unprecedented rate, with enormous economic growth accompanied by technological transformation. The economic and social structural changes have been associated with an individual’s unhealthy lifestyle [1]. A lifestyle consists of daily activities that could be considered healthy or unhealthy depending on individuals’ values or behaviors. Healthy lifestyle behaviors include sufficient physical activity, a balanced diet and nutrition, and spiritual growth [2]. Such unhealthy lifestyle behaviors have potentially contributed to a rise in lifestyle-related non-communicable diseases (NCDs) such as type 2 diabetes mellitus [3,4]. According to the International Diabetes Federation (IDF), Saudi Arabia is one of the countries worldwide with the highest prevalence of type 2 diabetes mellitus [5,6]. Cardinal determinant factors associated with this non-communicable disease, aside from the genetic predisposition, are modifiable factors such as physical inactivity, sedentary behaviors, and unhealthy eating patterns [6].

A number of investigations in Saudi Arabia reported the level of physical inactivity and unhealthy eating behaviors, such as the consumption of fatty animal products and refined sugars [7,8,9]. To date, the majority of studies about lifestyle factors conducted among the Saudi population show an increasing prevalence of predisposing factors such as obesity among Saudi women [7,8]. The prevalence of obesity among Saudi women who smoked was 40.23%, while overweight was 27%, and physical inactivity ranged from 53.2% to 98.1% [10]. Furthermore, over the last three years, there have been very positive socio-political changes in Saudi Arabia, which have resulted in more opportunities for Saudi women to become increasingly involved in many aspects of social life. The country mandated girls’ physical education in schools in 2017, allowed women to drive in 2018, and issued licenses to open women’s private gyms and fitness centers, thereby enabling women to take part in health-enhancing physical activity. These new policy changes that are happening in the country were highly commended, especially when it is known that the national prevalence of physical inactivity and sedentary behavior among Saudi women is exceedingly widespread [7,11,12]. In addition, the Saudi Arabian Ministry of Health’s New Initiatives and Saudi Vision 2030 highlighted the importance of healthy lifestyle habits in improving the health and welfare of all segments of the Saudi population, including women [13,14].

Some studies showed that unhealthy lifestyle behaviors potentially contributed to the prevalence of lifestyle-related NCDs in Saudi Arabia [3,4]. However, there is a scarcity of studies identifying whether women’s physical activity level, sedentary behavior, and sleep are independently associated with certain dietary habits. Understanding the inter-relationships between lifestyle factors in women actively engaging in physical activity is important for the development of effective promotional programs for healthy eating and active living. This would also greatly assist in developing national strategies for the promotion of healthy lifestyle behaviors and the prevention of health risks among the female population in this region. Therefore, this study was designed to investigate the level and associations of physical activity, sedentary behavior, sleep, and dietary habits among Saudi women attending fitness centers in Riyadh.

## 2. Materials and Methods

### 2.1. Study Design and Participants

In this descriptive cross-sectional study, all adult Saudi women aged 18 years and older without any physical disability were included. The ethical approval for the study was received from the Ethical Committee at Princess Nourah University (IRB Log Number: 18-0222).

### 2.2. Sampling and Data Gathering

All participants were randomly selected from 12 fitness centers in Riyadh city, Saudi Arabia, using clustered sampling technique stratified (geographic locations). Data gathering was conducted on random days on weekdays and weekends at the chosen fitness centers during 2019. Coordination with the management of fitness centers was properly observed to plan for data gathering. All females were approached during the visits and were asked to volunteer for the study. Written consent was obtained from each participant after explaining the study and their rights before distributing the questionnaire. The sample size needed for the study was calculated to be 384 participants. This was based on a sample proportion within 0.05 of the population proportion (0.50) with a 95% confidence level. A 20% was added to the sample for clustering effect and missing data, so the total target sample size became 460 participating females.

### 2.3. Anthropometric Measurement

Body weight was calculated at the nearest 100 g using a calibrated portable medical scale (Seca scale model 770, Seca, Hamburg, Germany), with minimal clothing and without shoes. Height was measured to the nearest 1 cm without shoes with the subject in full standing posture using calibrated portable measuring rod. The body mass index (BMI) was measured as kg body weight divided by height square in meters.

### 2.4. Survey/Measures

A two-part survey questionnaire was used to collect data from the participants. The first part includes information about the demographic characteristics of the participants. The second part was Arab Teen Lifestyle Study (ATLS) questionnaire that was used to gather lifestyle variables [11,15]. The questionnaire was previously shown to be valid and trustworthy for assessing physical activity and other lifestyle habits in adolescents as well as young adults [15,16]. The questionnaire collects information on the frequency, length, and intensity of physical activity with mild, moderate, and intense intensity over a typical week. The questionnaire covers different fields of activity, including transportation, domestic, fitness, and sporting, as well as leisure-time activities. The activity energy expenditure in metabolic equivalent of task (MET) during each type of PA was calculated using the compendium of physical activity [16]. In addition, total activity energy expenditure, as well as sums of moderate- and vigorous-intensity energy expenditure, were computed. In order to find the levels of physical activity of the participants, cut-off scores were used for the total activity energy expenditure in METs-minutes per week of 600 (4 METs of moderate-intensity activity multiplied by 150 min per week), 1500 (300 min per week multiply by 5 METs), and 3000 (420 min per week (60 min of daily physical activity) with a combination of moderate and vigorous-intensity physical activity.

The ATLS questionnaire includes questions related to sedentary behavior and sleep duration. Participants were informed about the average time spent on screen activities every day, including TV watching, video games, and computer and internet usage during weekdays and weekends. Participants also reported the average sleep lasts at night in hours spent on weekdays and weekends. The cut-off times used for the overall screen viewing time were below or above 3 h per day, while those used for the length of the sleep were below or above 7 h per day, as this number was defined as the minimum hours for normal regular adult sleep [17]. The dietary habits as frequency intakes of certain eating habits during a typical (usual) week were also part of the ATLS questionnaire. Participants mentioned how many days a week they eat breakfast, vegetables (cooked and uncooked), fruit, milk and dairy products, sugar-sweetened beverages (including soft drinks), fast foods, donuts/cakes, cookies, chocolates, and candies. A choice of responses was given to the participant ranging from zero intakes (never) to a maximum intake of 7 days per week (every day).

### 2.5. Data and Statistical Analysis

Data were entered, tested, cleaned, and analyzed in SPSS software, version 22 (IBM, Chicago, IL, USA). Descriptive statistics were calculated and presented as means and standard deviations (SD) or proportions. In addition, cross-tabulation with Chi-Square tests was used to calculate the frequency and proportion of selected variables. Independent t-tests were also used to test the differences between age categories in selected anthropometric variables. In addition, multivariable analysis (MANCOVA) of selected lifestyle variables while controlling for age and socio-demographic factors stratified by breakfast intake for less than five days per week versus five or more days per week. Further, we used multivariable analysis (three-way MANCOVA) of selected anthropometric and lifestyle variables while controlling for age and socio-demographic factors stratified by total activity energy expenditure levels in METs-min/week for the participants (below or above 50%ile), screen viewing time in hours per day as low (less than 3 h per day) and high (3 or more hours per day), and sleep duration in hours per night as insufficient (less than 7 h per night) and sufficient sleep (7 or more hours per night). The level of significance was set at *p* < 0.05.

## 3. Results

### 3.1. Anthropometric Measurements of Female Participants Attending Fitness Centers

Table 1 shows the anthropometric measurements of participating females. The sample was dichotomized based on age. Fifty-nine percent of the participants were less than 30 years old, and 40.1% were greater than 30 years old. The analysis found significant differences between the two groups in terms of age, weight, BMI, and combined prevalence of being overweight and obese. Overall, participants who were greater than 30 years old had higher BMIs and a higher prevalence of being overweight or obese than those less than 30 years old. The majority of female participants were unmarried (58.1%), had no children (63%), and had a college educational level (72.5%). Around 42% of married participants had at least one child. Moreover, most participants indicated that their parents’ highest attained educational level was “college”, with 23.7% of mothers and 43.7% of fathers achieving this level with significant (*p* < 0.001) differences for mother education level. Furthermore, 64.4% of the participants reported their family income between 10,000 and 30,000 SR per year.

### 3.2. Lifestyle Variables of Female Participants Attending Fitness Centers

**Figure 1:**
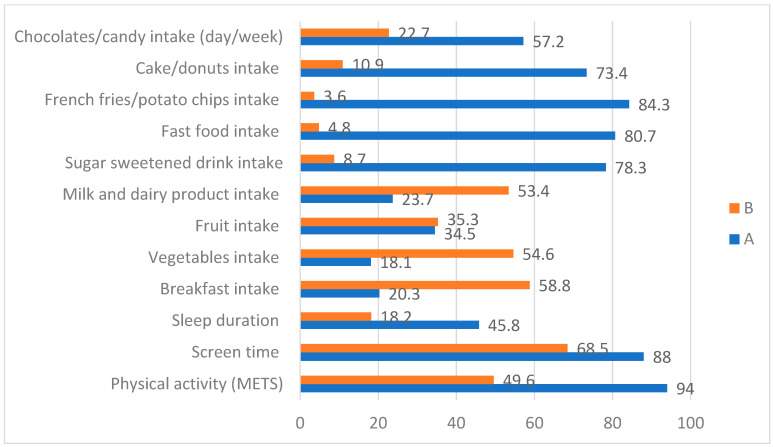
Proportion (%) of selected lifestyle variables at or above certain cut-off scores in female participants.

The proportion of selected lifestyle variables at or above certain cut-off scores in female participants is shown in Figure 1. The proportion of participants engaging in PA at a level exceeding energy expenditure of 600 METs-min/week (150 min per week of at least moderate-intensity physical activity) was high (94%); however, this proportion dropped to nearly 50% when the cut-off level for activity energy expenditure was set at 3000 METs-min/week, or an equivalent of one hour of daily PA with a combination of moderate and vigorous-intensity physical activity. Eighty-eight percent and 68.5% of participants reported screen time for more than 2 and 3 h per day, respectively. For daily sleep duration, 45.8 % of participants reported sleeping less than 7 h, and 18.2 % reported sleeping 8 or more hours per night. Except for fruit intake, those participants who consumed breakfast, vegetables, and milk and dairy products more than 5 days per week exceeded 50%. In addition, intakes of less healthy foods such as sugar-sweetened drinks, fast food, French fries/potato chips, cake/donuts, or chocolate/candy were at fairly minimal frequencies (Table 2).

### 3.3. Comparison of Selected Activity Energy Expenditure (MET-Minutes per Week) of Younger and Older Female Participants

The profile of selected physical activities in METs-minutes per week for the female participants is presented in Table 3. There were significant differences between the age of female participants in stair-stepping, household activity, dancing, vigorous-intensity sports, and the sum of all vigorous-intensity physical activity (*p* < 0.05).

### 3.4. Physical Activity Preferences among Female Participants

Table 4 illustrates the physical preferences among female participants. Most participants (73.3%) exercise in fitness centers, 45.1% usually exercise alone, and 37.3% exercise with friends/school mates. Moreover, almost half of the participants reported exercising in the afternoon (47.3%) compared to 26.1% in the evening and 6.5% in the morning. Reasons to exercise were maintaining health (26.4%), improving body shape (25%), and losing weight (24.7%).

### 3.5. Multivariable Analysis of Selected Lifestyle Variables Relative to Breakfast Intake Frequency and Socio-Demographic Factors

Table 5 displays the results of a multivariable analysis of selected lifestyle variables relative to breakfast intake frequency (less than five days per week versus five or more days per week) while controlling for age and socio-demographic factors. The analysis found having children or not, the purpose of losing weight, improving body shape, and completion are associated reasons for doing exercise by the Saudi females (*p* < 0.05). Significantly higher values were observed for the total physical activity energy expenditure in METs-min/week (*p* = 0.028) among females with higher breakfast intake (five or more days per week). Moreover, females with breakfast consumption five or more days per week showed higher intakes of vegetables (*p* < 0.001), fruit (*p* = 0.009), and milk/dairy products (*p* = 0.007), as well as a lower intake of French fries/potato chips (*p* = 0.001) compared with females with breakfast intake less than five days per week.

### 3.6. Multivariable Analysis of Selected Anthropometric and Lifestyle Variables While Controlling for Age and Socio-Demographic Factors

Table 6 shows the results of multivariable analysis (three-way MANCOVA) of selected anthropometric and lifestyle variables while controlling for age and socio-demographic factors stratified by total activity energy expenditure levels in METs-min/week for the participants (below or above 50%ile), screen viewing time in hours per day as low (less than 3 h per day) and high (3 or more hours per day), and sleep duration in hours per night as insufficient (less than 7 h per night) and sufficient sleep (7 or more hours per night). The findings indicate that there is no significant mean or interaction effect for the combined lifestyle factors on body weight or BMI. However, there are significant interaction effects for activity by sleep duration (*p* = 0.026) and sedentary time by sleep duration (*p* = 0.035) on fast food intake. As for the main effects of physical activity, there are significant effects on intakes of breakfast (*p* = 0.011), vegetables (*p* = 0.001), fruits (*p* < 0.001), sweetened sugar drinks (*p* = 0.022), fast foods (*p* = 0.002), and French fries/potato chips (*p* = 0.044). Screen time significantly affected intakes of fast foods (*p* = 0.009), French fries/potato chips (*p* = 0.008), and chocolates/candy (*p* = 0.001). In addition, there are significant main effects for sleep duration on breakfast intake (*p* = 0.028), vegetables intake (*p* = 0.004), milk/dairy products intake (*p* = 0.021), and sweetened sugar drinks intake (*p* = 0.018).

## 4. Discussion

The findings of the present study indicate that the females participating in attending fitness centers are generally very active; however, they have high proportions of obesity, sedentary behaviors, insufficient sleep, and less than optimal daily intakes of breakfast, vegetables, fruits, and milk/dairy products. In addition, there were significant clustering effects among healthy lifestyle habits. Furthermore, the analysis indicated that high PA levels with low screen time and sufficient sleep exhibited lower fast-food intake.

The current findings showed that the females in this study had a high level of PA, with the majority of them exceeding the current adults’ physical activity recommendations by the World Health Organization [18]. This indicates that participants were actively participating regularly in fitness centers. Private fitness centers in Saudi Arabia have been opening at an unprecedented rate in the last two years as a result of the new official governmental regulations that granted licenses to open private female gyms in the country. Earlier studies on PA showed that Saudi women were generally inactive [7,11,12]. Compared with previous studies, Saudi women were not actively participating in fitness centers [7,11,12]. The difference might be that there were limited fitness centers available in the past for Saudi females. In addition, the study shows significant differences between the age of female participants and their physical activities (e.g., stair-stepping, household activity, dancing, vigorous-intensity sports, and the sum of all vigorous-intensity physical activity). A previous study in Saudi Arabia indicated that moderate physical activity levels were more common than vigorous ones among Saudi women [19]. Although there were no significant differences in total activity energy expenditure between younger and older females in the present study, younger females had higher vigorous-intensity PA and were more engaged in dancing activities, while the older females were performing more household activities. This indicates that older females who are more likely to be married and have children prefer to engage in more household physical activities such as cooking, cleaning, vacuuming, and children’s care. In the United States, women tend to report participating in low to moderate physical activity [20]. Various studies also showed a general pattern of people becoming less physically active as they grow older [21,22].

Most participants in our study engaged in physical activity at fitness centers. This is in contrast with some earlier studies showing that most Saudi women engage in physical activity at home [23,24]. Maintaining health and losing weight was shown to be the main reasons why Saudi women exercised in previous studies [23,24]. In the present study, over 76% of the women exercised to maintain health, improve body shape or lose weight. Compared with previous studies, the main reason for most Saudi women engaging in physical activity and attending fitness centers in a study conducted in Hail, Saudi Arabia, was to lose weight and improve body image [25]. Interestingly, our study also showed that females with children are exercising significantly more for the purpose of losing weight, whereas females without children are more likely to exercise to improve body shape or in preparation for competition. These findings agree with another study that showed new mothers who exercised were seeking to lose weight [26]. Positive attitudes were associated with being active [27]. Interventions to improve physical activity might be successful in providing motivation, encouragement, and behavior modification among overweight and obese females. In addition, programs that focus on translating the intentions to perform more exercise among these individuals into actual behavior may help them to increase physical activity and stay active.

Active females in the present study were more likely to have favorable dietary habits, including a higher frequency of daily breakfast consumption. Similar findings related to the clustering of healthy and non-healthy behaviors were reported for Saudi adolescents, where physical activity was associated with healthful dietary habits while sedentary behaviors were independently linked to less healthy eating patterns [28]. Our results also agree with a recent study conducted on Saudi university females displaying a relationship between physical activity and healthy lifestyle factors [25,28]. The literature highlighted that health behaviors often coexist with a clear indication of clustering. In a group of university students from the State of Qatar, the clustering of physical activity, dietary habits, and BMI was found among both male and female students [29]. In addition, a previous study from Spain showed that risk factors such as smoking, diet, and physical inactivity tended to cluster among university students [30]. Moreover, among a large sample of a population aged 12 to 80 years from Southern China, breakfast eating habits were significantly related to healthy behavior and seemed to be a useful predictor of a healthy lifestyle [31]. In the present study, significant associations were found between females’ breakfast frequency and high levels of total physical activity and consumption of vegetables, fruit, and milk/dairy products.

In the present study, around more than half of the participants engage in sedentary activity for at least 3 h per day. A high prevalence of sedentary behavior was previously reported in Saudi women [23,32]. However, the high screen time reported in our study was concerning, given that the participants were physically active. This study also highlights that females with high screen time also reported significantly low activity levels and less healthy dietary habits. These findings are similar to previous research studies on young Saudi females showing that screen time was significantly related to several unfavorable dietary habits, whereas physical activity showed a significant correlation with eating fruit [33].

The findings of this study indicated that females with high screen time and insufficient sleep also exhibited other less healthy lifestyle behaviors. Recently, the associations between sedentary behavior and sleep duration, along with other risk factors, have attracted the attention of researchers [34,35]. These results are parallel with earlier studies associating screen time with insufficient and low-quality sleep [36]. Sleep and physical activity are interrelated and involve multiple physiological and psychological pathways [37,38]. The present findings revealed that high screen time and/or insufficient sleep are significantly associated with unhealthy lifestyle habits, including skipping breakfast, low consumption of fruits and vegetables, and high intake of fast foods. Previous local research on young Saudi females showed that poor sleep quality was significantly associated with several lifestyle factors, including low physical activity and low intake of breakfast [29,39]. In addition, data from the National Health and Nutrition Survey, which examined sleep duration and intake of sugar-sweetened beverages from two 24 h dietary recall among 18,779 US adults, indicated that short sleep is associated with greater intake of sugared caffeinated sodas, a relationship that may have important, though unrecognized, implications on physical health [40]. Furthermore, another nationwide representative survey among Japanese adolescents confirmed the association between unhealthy dietary behaviors and sleep disturbances [41].

In terms of dietary habits, our study showed that a higher intake of fruits and vegetables, lower consumption of sweetened-sugar drinks, fast foods, French fries/potato chips, and chocolates/candy were associated with lower levels of physical activity. Our results are in line with a previous study among Saudi adolescents in which diet indicators such as vegetable and fruit consumption were significantly correlated with physical activity [11]. In another study, no significant association was found between dietary patterns and the majority of women who had low to no physical activity [42]. Similarly, skipping breakfast was associated with reduced levels of physical activity. A previous study reported similar associations [43]. In addition, Croll et al. reported that adolescents playing sports ate healthier and more nutritious food than their peers who do not play sports [44]. These differing findings may be explained by the fact that more than half of the participants were overweight and obese. It is worth noting that identifying the interrelationships between these factors can help develop effective lifestyle interventions and strategies.

## 5. Conclusions

The current study has some strengths and limitations. This study can be a valuable addition to the local literature on the engagement of Saudi women in physical activity and the interrelationships of physical activity, sedentary behavior, sleep, and dietary habits among females attending fitness centers. The sample was a representative of females attending fitness centers in Riyadh, a metropolitan city that encompassed people from all parts of the country. The response rate in this study is considerably high. Further, the study’s instrument was validated previously. The analysis of the data controlled for possible confounding factors. As for the limitations, first, the study was cross-sectional, which limits any deduction of causality. Second, participants reported the frequency of their dietary intake regardless of quantities or portion size, which may have influenced the association between dietary intake with other variables. In addition, there may be other variables that were not measured (example: type of diet, number of calories, blood pressure, and glucose serum) which could interfere with the lifestyle and physical fitness of the participants. Third, the physical activity questionnaire used in this study, though comprehensive, valid, and reliable for younger age females (less than 30 years), has not been validated for an older age group (above 30 years of age). Forth, the sample was drawn from female participants in fitness centers, which may not exactly generalize the findings to those females not attending fitness centers.

The present study investigated the levels of physical activity, sedentary behaviors, sleep duration, and dietary habits and examined the associations among these variables in a representative sample of Saudi women attending fitness centers in Riyadh. The findings showed that more than half of the participants were either overweight or obese. Overall, the participants were highly active, exceeding the recommended physical activity needed to enhance health. However, despite having high physical activity levels, the Saudi females showed a cluster of unhealthy lifestyle habits, including high screen time, insufficient nocturnal sleep, and inadequate intake of breakfast, fruit, and vegetables. In addition, younger-age females displayed higher engagement in vigorous physical activity than the younger-age group. Moreover, those females with good breakfast habits (five or more intakes per week) exhibited other good lifestyle behaviors, such as a higher intake of fruit and vegetables and increased levels of physical activity. Moreover, high screen time and/or insufficient sleep are significantly associated with unhealthy lifestyle habits, including skipping breakfast, low consumption of fruits and vegetables, and high intake of fast foods. In conclusion, our findings confirmed the clustering of healthy eating habits with increased physical activity levels and the association of unhealthy eating habits with high sedentary behaviors and insufficient sleep in Saudi females.

## Figures and Tables

**Table 1:** Anthropometric measurements of the participating females (means and standard deviations).

Variable	All (N = 458)	<30 Years Old (59.9%)	30+ Years Old (40.1%)	*p*-Value *
Age (years)	29.2 (8.2)	23.8 (3.4)	37.1 (6.5)	-
Body weight (kg)	69.4 (14.3)	66.4 (13.8)	73.7 (14.9)	<0.001
Height (cm)	158.7 (5.5)	158.6 (5.2)	158.8 (6.0)	0.711
Body mass index (BMI)	27.5 (5.4)	26.4 (5.3)	29.2 (5.6)	<0.001
Overweight or obese (%)	62.6	53.3	76.4	<0.001

* *t*-tests for independent samples for the differences between age groups and Chi-Square tests for the proportion.

**Table 2:** Proportion (%) of selected lifestyle variables at or above certain cut-off scores in female participants.

Variable	Cut-Offs	Proportion (%)
PA (METs-min/week)	>600	94.0
	>1500	82.0
	>3000	49.6
Screen time (hours/day)	>2 h	88.0
	>3 h	68.5
Sleep duration (hours/night)	7+ h	45.8
	8+ h	18.2
Breakfast intake (day/week)	1–2 days	20.3
	3–4 days	10.9
	5+ days	58.8
Vegetables intake (day/week)	1–2 days	18.1
	3–4 days	27.3
	5+ days	54.6
Fruit intake (day/week)	1–2 days	34.5
	3–4 days	30.2
	5+ days	35.3
Milk and dairy product intake (day/week)	1–2 days	23.7
	3–4 days	22.9
	5+ days	53.4
Sugar-sweetened drink intake(day/week)	1–2 days	78.3
	3–4 days	13.0
	5+ days	8.7
Fast food intake (day/week)	1–2 days	80.7
	3–4 days	14.5
	5+ days	4.8
French fries/potato chips intake (day/week)	1–2 days	84.3
	3–4 days	12.1
	5+ days	3.6
Cake/donuts intake (day/week)	1–2 days	73.4
	3–4 days	15.7
	5+ days	10.9
Chocolates/candy intake (day/week)	1–2 days	57.2
	3–4 days	20.0
	5+ days	22.7

Note: Cutoffs: PA (METs-min/week) a. >600, b. >3000; Screen time (hours/day) a. >2 h, b. >3 h; Sleep duration (hours/night) a. 7+ hours, b. 8+ hours; breakfast intake, vegetable intake, milk and dairy product intake, sugar sweetened drink, fast food intake, French fries/potato chip intake, cake donuts intake, chocolate/candy intake (day/week), a. 1–2 days, b. 5+ days. MET = Metabolic equivalent of task. 1 MET = energy expenditure at rest.

**Table 3:** Means and standard deviations of selected activity energy expenditure (MET-minutes per week) of younger and older female participants.

Variable	All (N = 460)	<30 Years Old (59.9%)	30+ Years Old (40.1%)	*p* Value *
Walking (METs-min/week) **	576.1 (629.6)	607.9 (624.1)	550.1 (694.5)	0.378
Stair Stepping (METs-min/week)	92.9 (51.9)	98.1 (58.5)	86.7 (41.7)	0.031
Jogging (METs-min/week)	525.2 (817.5)	538.8 (737.7)	490.3 (926.5)	0.555
Cycling (METs-min/week)	346.1 (513.1)	320.7 (514.7)	377.9 (536.6)	0.277
Swimming (METs-min/week)	208.6 (540.9)	157.1 (420.4)	268.3 (687.9)	0.064
Martial art (METs-min/week)	54.3 (197.4)	66.4 (235.1)	40.5 (140.8)	0.163
Resistance training (METs-min/week)	496.4 (645.7)	553.6 (721.4)	446.6 (544.3)	0.104
Household (METs-min/week)	428.3 (809.2)	292.2 (539.9)	615.3 (1073.7)	<0.001
Dancing (METs-min/week)	299.7 (574.1)	350.8 (586.2)	199.5 (465.6)	0.006
Moderate-intensity sports (METs-min/week)	218.6 (614.1)	239.8 (670.4)	159.3 (425.8)	0.136
Vigorous-intensity sports (METs-min/week)	479.5 (003.8)	557.5 (1021.5)	360.6 (711.8)	0.021
Sum of all moderate-intensity physical activity (METs-min/week)	1512.7 (1361.8)	1408.0 (1232.1)	1633.2 (1527.8)	0.114
Sum of all vigorous-intensity PA(METs-min/week)	2212.9 (2154.2)	2374.9 (2295.7)	1961.8 (1935.7)	0.049
Total PA energy expenditure (METs-min/week)	3725.7 (2748.8)	3782.9 (2769.1)	3595.0 (2735.7)	0.496

* *t*-tests for independent samples for the differences between age groups. ** MET = metabolic equivalent of task.

**Table 4:** Physical activity preferences among female participants.

Variable (N)	Frequency	%
Where do you mostly exercise?		
Fitness center	294	73.3
Sport/recreational center	74	18.5
At home	23	5.7
At university	6	1.5
Other than the above	4	1.0
Total	401	100
*p* value *	0.647	
With whom do you usually exercise?		
Alone	181	45.1
With a friends/school mates	150	37.3
With relatives	27	6.7
With parents	13	3.2
Other than the above	31	7.7
Total	402	100
*p* value *	0.094	
When do you mostly exercise?		
Afternoon	190	47.3
Evening	105	26.1
Morning	26	6.5
No specific time	81	20.1
Total	402	100
*p* value *	0.120	
Reason for being active		
For health	248	26.4
To improve body shape	235	25.0
For weight loss	233	24.7
For recreation	142	15.1
For competition	54	5.7
For meeting friends	29	3.1
Total (more than one choice is allowed)	941	100
*p* value *	<0.001	

* Chi-square test for the differences between observed and expected frequencies.

**Table 5:** Multivariable analysis (MANCOVA) of selected lifestyle variables relative to breakfast intake frequency (less than five days per week versus five or more days per week) while controlling for age and socio-demographic factors.

Variable	Breakfast Intake		*p*-Value *
	<5 Day/Week 1.63 (1.4)	5+ Days/Week 6.6 (0.74)	
Number of participants	149	225	-
Body weight (kg)	70.0 (15.5)	69.2 (14.5)	0.480
Body mass index	27.8 (5.8)	27.5 (5.6)	0.553
Total PA energy expenditure (MET-min/week)	3381.1 (2516.2)	3991.0 (2777.1)	0.028
Sum of all moderate-intensity physical activity (MET-min/week)	1389.6 (1252.4)	1610.9 (1379.4)	0.128
Sum of all vigorous-intensity PA (MET-min/week)	1991.5 (1921.4)	2380.2 (2233.2)	0.065
Average screen time (hours/day)	4.49 (2.2)	4.37 (2.2)	0.513
Average sleep duration (hours/night)	6.63 (1.6)	6.82 (1.5)	0.230
Vegetables intake (day/week)	4.25 (2.2)	5.04 (1.9)	<0.001
Fruit intake (day/week)	3.38 (2.2)	4.02 (2.2)	0.009
Milk/dairy products intake (day/week)	4.30 (2.4)	4.84 (2.3)	0.034
Sweetened sugar drinks intake (day/week)	1.70 (1.9)	1.40 (1.8)	0.166
Fast foods intake (day/week)	1.77 (1.5)	1.49 (1.4)	0.084
French fries/potato chips intake (day/week)	1.60 (1.5)	1.12 (1.3)	0.001
Cake/donuts intake (day/week)	2.09 (1.9)	1.85 (1.8)	0.238
Chocolates/candy intake (day /week)	2.83 (2.3)	2.73 (2.3)	0.685

* Wilks’ Lambda *p* values: age < 0.001; education level = 0.004; family income = 0.030; and breakfast intake < 0.001. Data are means and standard deviations.

**Table 6:** Multivariable analysis (three-way MANCOVA) of selected anthropometric and lifestyle variables while controlling for age and socio-demographic factors stratified by total activity energy expenditure levels in METs-min/week for the participants (below or above 50%ile), screen viewing time in hours per day as low (less than 3 h per day) and high (3 or more hours per day), and sleep duration in hours per night as insufficient (less than 7 h per night) and sufficient sleep (7 or more hours per night).

Variable	Screen Time	Sleep Duration	Activity Level (METs-min/Week)		*p*-Value *
			Low Active 1718.9 (838.4)	High Active 5715.1 (2559.5)	
Body weight (kg)	Low	Insufficient	69.2 (12.3)	73.7 (17.9)	Activity level: 0.779 Screen time: 0.743 Sleep: 0.440 Activity by screen:0.891 Activity by sleep: 0.505 Screen by sleep:0.873 Activity by screen by sleep interactions: 0.060
		Sufficient	72.2 (9.9)	68.8 (13.5)	
	High	Insufficient	68.9 (15.9)	67.4 (15.1)	
		Sufficient	69.6 (14.0)	69.6 (16.1)	
Body mass index	Low	Insufficient	27.8 (5.5)	29.0 (7.0)	Activity level: 0.536 Screen time: 0.427 Sleep: 0.431 Activity by screen: 0.993 Activity by sleep: 0.704 Screen by sleep: 0.870 Activity by screen by sleep interactions: 0.177
		Sufficient	28.8 (4.9)	27.8 (5.2)	
	High	Insufficient	27.4 (5.7)	26.8 (5.5)	
		Sufficient	27.6 (5.5)	27.3 (5.7)	
Total PA energy expenditure (MET-min/week)	Low	Insufficient	1873.5 (841.9)	5823.9 (2647.7)	Activity level: < 0.001 Screen time: 0.022 Sleep: 0.178 Activity by screen:0.141 Activity by sleep: 0.065 Screen by sleep: 0.954 Activity by screen by sleep interactions: 0.964
		Sufficient	1905.6 (691.9)	6471.2 (2609.6)	
	High	Insufficient	1791.2 (835.4)	5076.5 (2068.1)	
		Sufficient	1648.6 (791.1)	5782.6 (2667.4)	
Sum of all moderate-intensity physical activity (MET-min/week)	Low	Insufficient	776.3 (669.5)	2379.4 (1501.2)	Activity level: < 0.001 Screen time: 0.163 Sleep: 0.924 Activity by screen: 0.184 Activity by sleep: 0.739 Screen by sleep: 0.200 Activity by screen by sleep interactions: 0.550
		Sufficient	834.5 (512.3)	2577.7 (1584.6)	
	High	Insufficient	820.7 (560.0)	2222.8 (1484.7)	
		Sufficient	737.6 (574.5)	2000.0 (1351.2)	
Sum of all vigorous-intensity PA(MET-min/week)	Low	Insufficient	1097.2 (771.9)	3444.6 (2180.5)	Activity level: < 0.001 Screen time: 0.117 Sleep: 0.166 Activity by screen: 0.0468 Activity by sleep: 0.077 Screen by sleep: 0.442 Activity by screen by sleep interactions: 0.663
		Sufficient	1071.2 (717.2)	3893.5 (2258.9)	
	High	Insufficient	970.5 (620.0)	2853.7 (2116.4)	
		Sufficient	911.1 (672.8)	3782.7 (2612.7)	
Average screen time (hours/day)	Low	Insufficient	2.17 (0.67)	2.31 (0.50)	Activity level: 0.363 Screen time: < 0.001 Sleep: 0.601 Activity by screen: 0.939 Activity by sleep: 0.366 Screen by sleep: 0.957 Activity by screen by sleep interactions: 0.355
		Sufficient	2.45 (0.53)	2.39 (0.52)	
	High	Insufficient	5.08 (1.8)	5.57 (2.1)	
		Sufficient	5.46 (1.7)	5.4 (2.1)	
Average sleep duration (hours/night)	Low	Insufficient	5.54 (1.1)	5.43 (0.98)	Activity level: 0.461 Screen time: 0.078 Sleep: < 0.001 Activity by screen: 0.455 Activity by sleep: 0.701 Screen by sleep: 0.656 Activity by screen by sleep interactions: 0.575
		Sufficient	8.04 (0.90)	7.78 (0.62)	
	High	Insufficient	5.67 (0.98)	5.63 (1.0)	
		Sufficient	8.150. (77)	8.18 (0.80)	
Breakfast intake (day/week)	Low	Insufficient	4.42 (2.7)	5.22 (2.6)	Activity level: 0.011 Screen time: 0.269 Sleep: 0.028 Activity by screen: 0.320 Activity by sleep: 0.688 Screen by sleep: 0.087 Activity by screen by sleep interactions: 0.309
		Sufficient	4.84 (2.3)	4.96 (2.7)	
	High	Insufficient	3.50 (2.6)	4.35 (2.9)	
		Sufficient	4.50 (2.7)	5.68 (2.1)	
Vegetables intake (day/week)	Low	Insufficient	4.5 (2.2)	5.8 (1.9)	Activity level: 0.001 Screen time: 0.682 Sleep: 0.004 Activity by screen: 0.508 Activity by sleep: 0.221 Screen by sleep: 0.208 Activity by screen by sleep interactions: 0.597
		Sufficient	4.32 (2.3)	5.56 (2.1)	
	High	Insufficient	4.03 (2.1)	4.36 (2.1)	
		Sufficient	4.66 (2.0)	5.58 (1.8)	
Fruit intake (day/week)	Low	Insufficient	3.31 (2.2)	4.69 (2.1)	Activity level: < 0.001 Screen time: 0.382 Sleep: 0.159 Activity by screen: 0.516 Activity by sleep: 0.622 Screen by sleep: 0.237 Activity by screen by sleep interactions: 0.605
		Sufficient	3.37 (2.1)	4.44 (2.3)	
	High	Insufficient	2.91 (1.9)	3.77 (2.3)	
		Sufficient	3.61 (2.3)	4.34 (2.2)	
Milk/dairy products intake (day/week)	Low	Insufficient	4.33 (2.3)	4.47 (2.3)	Activity level: 0.669 Screen time: 0.437 Sleep: 0.021 Activity by screen: 0.456 Activity by sleep: 0.733 Screen by sleep: 0.373 Activity by screen by sleep interactions: 0.424
		Sufficient	5.37 (2.2)	5.00 (2.2)	
	High	Insufficient	4.31 (2.3)	4.50 (2.4)	
		Sufficient	4.59 (2.3)	4.98 (2.3)	
Sweetened sugar drinks intake (day/week)	Low	Insufficient	1.58 (1.9)	1.36 (1.6)	Activity level: 0.022 Screen time: 0.252 Sleep: 0.018 Activity by screen: 0.757 Activity by sleep: 0.204 Screen by sleep: 0.773 Activity by screen by sleep interactions: 0.487
		Sufficient	1.63 (1.9)	0.67 (87)	
	High	Insufficient	2.08 (2.2)	1.70 (2.1)	
		Sufficient	1.56 (1.8)	1.10 (1.5)	
Fast foods intake (day/week)	Low	Insufficient	0.89 (0.62)	1.25 (1.4)	Activity level: 0.002 Screen time: 0.009 Sleep: 0.833 Activity by screen: 0.088 Activity by sleep: 0.026 Screen by sleep: 0.035 Activity by screen by sleep interactions: 0.149
		Sufficient	1.95 (1.8)	1.04 (0.81)	
	High	Insufficient	2.27 (1.4)	1.64 (1.3)	
		Sufficient	1.97 (1.67)	1.26 (1.2)	
French fries/potato chips intake (day/week)	Low	Insufficient	0.83 (0.85)	0.89 (1.1)	Activity level: 0.044 Screen time: 0.008 Sleep: 0.507 Activity by screen: 0.134 Activity by sleep: 0.129 Screen by sleep: 0.188 Activity by screen by sleep interactions: 0.650
		Sufficient	1.26 (1.3)	0.89 (93)	
	High	Insufficient	1.77 (1.5)	1.50 (1.4)	
		Sufficient	1.70 (1.7)	0.98 (1.1)	
Cake/donuts intake (day/week)	Low	Insufficient	1.69 (1.5)	2.17 (2.0)	Activity level: 0.112 Screen time: 0.172 Sleep: 0.058 Activity by screen: 0.322 Activity by sleep: 0.159 Screen by sleep: 0.983 Activity by screen by sleep interactions: 0.279
		Sufficient	1.79 (1.4)	1.30 (1.5)	
	High	Insufficient	2.42 (2.1)	2.03 (1.9)	
		Sufficient	2.08 (1.8)	1.58 (1.7)	
Chocolates/candy intake (day/week)	Low	Insufficient	2.11 (2.0)	2.39 (2.0)	Activity level: 0.112 Screen time: 0.001 Sleep: 0.342 Activity by screen: 0.369 Activity by sleep: 0.209 Screen by sleep: 0.277 Activity by screen by sleep interactions: 0.227
		Sufficient	2.53 (1.9)	1.96 (1.9)	
	High	Insufficient	3.64 (2.3)	2.92 (2.4)	
		Sufficient	3.13 (2.3)	2.39 (2.3)	

* Wilks’ Lambda *p* values: age < 0.001; education level = 0.028; family income = 0.077; activity level < 0.001; screen time < 0.001; sleep duration < 0.001; activity level by screen time interactions = 0.478; activity level by sleep duration interactions 0.679; screen time by sleep duration interactions = 0.506; activity level by screen time by sleep duration interactions = 0.283. Data are means and standard deviations.

## Data Availability

The data set used is locked and stored in the College of Applied Medical Science at King Saud University and can be obtained from the principal investigator on reasonable request.
